# Supplementary material for: A Critical View of the Application of the APEX Software (Aqueous Photochemistry of Environmentally-Occurring Xenobiotics) to Predict Photoreaction Kinetics in Surface Freshwaters
Source: Molecules. 2019 Dec 18;25(1):9. doi: 10.3390/molecules25010009 (PMC7017383; doi:10.3390/molecules25010009)
Supplement: Supplementary file 1 [file molecules-25-00009-s001.zip › 3.2.4_gcc-4.4.0.zip!/3.2.4_gcc-4.4.0/3.2.4_gcc-4.4.0/doc/octave/html/interpreter/Axes-Properties.html]

Axes Properties - Untitled


Next: Line Properties,
Previous: Figure Properties,
Up: Graphics Object Properties


---

##### 15.2.2.3 Axes Properties

`position`: A vector specifying the position of the plot, excluding titles, axes and legend. The four elements of the vector are the coordinates of the lower left corner and width and height of the plot, in units normalized to the width and height of the plot window. For example, `[0.2, 0.3, 0.4, 0.5]` sets the lower left corner of the axes at (0.2, 0.3) and the width and height to be 0.4 and 0.5 respectively. See also the `outerposition` property. `title`: Index of text object for the axes title. `box`: Either `"on"` or `"off"` to toggle display of the box around the axes. `key`: Either `"on"` or `"off"` to toggle display of the legend. Note that this property is not compatible with matlab and may be removed in a future version of Octave. `keybox`: Either `"on"` or `"off"` to toggle display of a box around the legend. Note that this property is not compatible with matlab and may be removed in a future version of Octave. `keypos`: An integer from 1 to 4 specifying the position of the legend. 1 indicates upper right corner, 2 indicates upper left, 3 indicates lower left, and 4 indicates lower right. Note that this property is not compatible with matlab and may be removed in a future version of Octave. `dataaspectratio`: A two-element vector specifying the relative height and width of the data displayed in the axes. Setting `dataaspectratio` to 1, 2] causes the length of one unit as displayed on the y-axis to be the same as the length of 2 units on the x-axis. Setting `dataaspectratio` also forces the `dataaspectratiomode` property to be set to `"manual"`. `dataaspectratiomode`: Either `"manual"` or `"auto"`. `xlim` `ylim` `zlim` `clim`: Two-element vectors defining the limits for the x, y, and z axes and the Setting one of these properties also forces the corresponding mode property to be set to `"manual"`. `xlimmode` `ylimmode` `zlimmode` `climmode`: Either `"manual"` or `"auto"`. `xlabel` `ylabel` `zlabel`: Indices to text objects for the axes labels. `xgrid` `ygrid` `zgrid`: Either `"on"` or `"off"` to toggle display of grid lines. `xminorgrid` `yminorgrid` `zminorgrid`: Either `"on"` or `"off"` to toggle display of minor grid lines. `xtick` `ytick` `ztick`: Setting one of these properties also forces the corresponding mode property to be set to `"manual"`. `xtickmode` `ytickmode` `ztickmode`: Either `"manual"` or `"auto"`. `xticklabel` `yticklabel` `zticklabel`: Setting one of these properties also forces the corresponding mode property to be set to `"manual"`. `xticklabelmode` `yticklabelmode` `zticklabelmode`: Either `"manual"` or `"auto"`. `xscale` `yscale` `zscale`: Either `"linear"` or `"log"`. `xdir` `ydir` `zdir`: Either `"forward"` or `"reverse"`. `xaxislocation` `yaxislocation`: Either `"top"` or `"bottom"` for the x-axis and `"left"` or `"right"` for the y-axis. `view`: A three element vector specifying the view point for three-dimensional plots. `visible`: Either `"on"` or `"off"` to toggle display of the axes. `nextplot`: May be one of `"new"` `"add"` `"replace"` `"replacechildren"` `outerposition`: A vector specifying the position of the plot, including titles, axes and legend. The four elements of the vector are the coordinates of the lower left corner and width and height of the plot, in units normalized to the width and height of the plot window. For example, `[0.2, 0.3, 0.4, 0.5]` sets the lower left corner of the axes at (0.2, 0.3) and the width and height to be 0.4 and 0.5 respectively. See also the `position` property.
